# Exploring the performance reserve: Effect of different magnitudes of power output deception on 4,000 m cycling time-trial performance

**DOI:** 10.1371/journal.pone.0173120

**Published:** 2017-03-09

**Authors:** Mark R. Stone, Kevin Thomas, Michael Wilkinson, Emma Stevenson, Alan St. Clair Gibson, Andrew M. Jones, Kevin G. Thompson

**Affiliations:** 1 Centre for Human Performance, Health and Wellbeing, Buckinghamshire New University, High Wycombe, United Kingdom; 2 Faculty of Health & Life Sciences, Northumbria University, Newcastle upon Tyne, United Kingdom; 3 Institute of Cellular Science, Newcastle University, Newcastle upon Tyne, United Kingdom; 4 School of Health, Sport and Human Performance, University of Waikato, Hamilton, New Zealand; 5 School of Sport and Health Sciences, University of Exeter, Exeter, United Kingdom; 6 Research Institute for Sport and Exercise (UCRISE), University of Canberra, Canberra, Australia; University of Alabama at Birmingham, UNITED STATES

## Abstract

**Purpose:**

The aim of the present study was to investigate whether a magnitude of deception of 5% in power output would lead to a greater reduction in the amount of time taken for participants to complete a 4000 m cycling TT than a magnitude of deception of 2% in power output, which we have previously shown can lead to a small change in 4000 m cycling TT performance.

**Methods:**

Ten trained male cyclists completed four, 4000 m cycling TTs. The first served as a habituation and the second as a baseline for future trials. During trials three and four participants raced against a pacer which was set, in a randomized order, at a mean power output equal to 2% (+2% TT) or 5% (+5% TT) higher than their baseline performance. However participants were misled into believing that the power output of the pacer was an accurate representation of their baseline performance on both occasions. Cardiorespiratory responses were recorded throughout each TT, and used to estimate energy contribution from aerobic and anaerobic metabolism.

**Results:**

Participants were able to finish the +2% TT in a significantly shorter duration than at baseline (*p* = 0.01), with the difference in performance likely attributable to a greater anaerobic contribution to total power output (*p* = 0.06). There was no difference in performance between the +5% TT and +2% TT or baseline trials.

**Conclusions:**

Results suggest that a performance reserve is conserved, involving anaerobic energy contribution, which can be utilised given a belief that the exercise will be sustainable however there is an upper limit to how much deception can be tolerated. These findings have implications for performance enhancement in athletes and for our understanding of the nature of fatigue during high-intensity exercise.

## Introduction

There is general agreement that the pacing strategy that athletes adopt during a closed loop, self-paced cycling time-trial (TT) is regulated in an anticipatory manner [1]. The rate of increase in the conscious perception of exertion (RPE) is evaluated in relation to a pre- exercise template, and any mismatch between the two results in a modification to exercise intensity [1]. As such, it has been suggested that athletes operate within a metabolic reserve [2–4]. The template RPE is thought to be derived from previous experiences of completing trials of a similar duration, whereas the magnitude of the metabolic reserve is thought to be related to the athlete’s confidence that the TT can be completed without negative consequences arising [5].

Manipulating an athlete’s feedback could therefore distort the amount of effort they are able to attribute to an exercise challenge, and ultimately affect performance. We have previously shown that it was indeed possible to reduce the amount of time taken to complete a 4000 m cycling TT by misleading participants into believing they were exercising at the same intensity as in a previous trial, when in fact the power output was 2% greater [6]. Several other researchers have conducted similar experiments examining the effects of deception during endurance exercise performance [4, 7–10]. However, the results of these previous studies are equivocal, with findings ranging from improvements in performance under conditions of deception [8, 10], no effect of deception on performance [7, 9] and impaired performance under conditions of deception [4].

It is difficult to compare the findings of these previous studies due to differences in experimental design. For example, the duration of the exercise bout varies and therefore the proportion and distribution of the anaerobic and aerobic energy contribution will be different. In addition, whilst two studies investigated the effects of deception on self-paced TT performance [4, 7], another two reported changes in time to volitional exhaustion during cycling at a constant power output [8, 9]; and the nature of deception has ranged from manipulation of clock calibration [8], false speed feedback [7], knowledge of exercise intensity [9] and inaccurate comparative feedback on whether participants are winning or losing, compared to their baseline trial [6]. Finally some of these studies have been limited by small sample sizes [6, 9, 10], the use of untrained participants [8, 9] and the use of exercise tests which have been shown to have low test-retest reliability [8,9].

For self-paced cycling TTs, deception has been shown to result in improvements in performance [6] and no change [7]. The differences in the results between these two studies could be due to differences in the magnitude of deception. In our previous study [7] the power output of the pacer in the deception condition was 2% greater than power output at baseline and was the smallest increase in power output that had previously been shown to represent a meaningful change in performance [11]. This equated to an average increase of approximately 6 W over a 4000 m trial, which participants were able to exceed. In Micklewright et al. [7], participants completed two baseline 20-km TTs during which the speed shown was 5% greater than their actual speed, and a third trial, in which accurate speed feedback was provided. Although speed and power output are not linearly related, modelling has shown that a 1.0% increase in speed is similar to a 2.9% increase in mean power output [12].The power output at baseline in the study by Micklewright et al. [7] was 259 W, therefore participants would have had to sustain an additional mean power output of approximately 38 W (14.7%) in order to match their expected performance over a distance of 20 km, which is also markedly longer than the trials in our previous study (4000 m). Given these circumstances, it is unlikely that such a difference in speed would have escaped detection by participants in the Micklewright et al. [7] study. Nevertheless, it is possible that the maximum tolerable magnitude of deception during cycling lies somewhere between that assessed by Stone et al. [6] and that evaluated by Micklewright et al. [7]. Further research is warranted to determine the magnitude of the tolerable deception that enhances performance.

The aim of the present study was therefore to investigate whether a magnitude of deception of 5% in power output would lead to a greater reduction in the amount of time taken for participants to complete a 4000 m cycling TT than a magnitude of deception of 2% in power output, which we have previously shown can lead to a small change in 4000 m cycling TT performance [6, 11].

## Methods

### Participants

Ten male cyclists (age, 31.4 ± 6.3 y, stature, 1.8 ± 0.7 m, mass, 79.5 ± 8.3 kg, $\dot{V}O_{2\mathrm{peak}}$ 4.88 ± 0.3 L· min-1) who regularly performed cycling training and time trial competitions volunteered to take part in this study. Participants were instructed to maintain their normal diet throughout the experiment and to refrain from strenuous exercise and the consumption of caffeine or alcohol in the 24 hours preceding each laboratory testing session. All participants provided written informed consent before taking part in the study, which was approved by the research ethics committee of Northumbria University, UK.

### Experimental design

Participation involved a total of five visits to the laboratory; a preliminary visit, a practice 4000 m TT and three experimental 4000 m TTs. The practice TT served to familiarize participants with all procedures and equipment and the demands of the trial [11]. Performance in the first experimental 4000 m TT was used as a baseline (BL). During the remaining two trials participants were informed that they would be racing against a computer-generated avatar that represented their BL performance, and that the aim of the study was to examine the consistency of performance during repeated cycling TTs. However, during one of these TTs (+2% TT) the mean power output of the avatar was actually equal to 102% of BL, and during the other TT (+5% TT) the mean power output of the avatar was set at 105% of BL. Participants were debriefed as to the true nature of the experiment on completion of the study. The difference in power output between BL and the avatar during +2% and +5% are quantifiable as small and medium effects using Cohen’s effect size categories [13]. The order in which participants were exposed to the +2% and +5% conditions was counter balanced in order to minimize the potential for training or habituation-induced bias. Intra-participant testing was conducted at the same time of day in order to minimize the confounding effects of circadian variations. Three to seven days separated test sessions. Each participant used the same electromagnetically braked cycle ergometer (Velotron, Racermate, Seattle) and personal bike set-up for each session. Prior to each visit participants were asked to refrain from strenuous exercise (for at least 24 h) and caffeine (for at least 12 h) and to arrive in a fully rested, hydrated state. Participants completed a 24-h food diary before the practice TT and were instructed to replicate their intake as closely as possible before each subsequent trial.

### Procedures

#### Preliminary visit

Participants first completed a sub-maximal incremental test that started at 150 W and increased by 25 W every 4 min. Heart rate (Polar Electro, Finland) and $\dot{V}O_{2}$ (Cortex Metalyser 3b, Biophysik, Germany) were recorded continuously throughout the test and data were averaged during the final minute of each stage. Rating of perceived exertion and blood lactate concentration (B[La], Biosen C_Line, EKF diagnostic, Barleben, Germany) were recorded during the final 30-s of each stage. Blood lactate concentrations were plotted against power output at each stage and the exercise intensities at which B [La] increased by 1 mmol·l^-1^ from resting and which corresponded to a concentration of 4 mmol·l^-1^ were expressed as LT_1_ and LT_2_, respectively. The submaximal exercise test was terminated after LT_2_ had been identified.

After the completion of the submaximal exercise test, participants were given 5 min to rest before the commencement of the maximal exercise test. Subsequently, participants were asked to select their preferred cadence, and then the resistance was adjusted to 200 W and increased by 5 W every 15 s. The test was terminated when cadence reduced by more than 15% or when participants felt that they could no longer sustain the required exercise intensity. The highest $\dot{V}O_{2}$ averaged over a 30 s period was defined as $\dot{V}O_{2\mathrm{peak}}$. The linear relationship between the power output and $\dot{V}O_{2}$ that was measured during each stage of the submaximal exercise test was extrapolated for the estimation of Pmax.

#### 4000 m self-paced time-trials

Participants completed a practice 4000 m TT, and three experimental 4000 m TTs on an electromagnetically braked cycle ergometer (Velotron, Racermate, Seattle) interfaced with Velotron 3D software. The 3D software supports the instantaneous generation of an on-screen avatar that illustrates the cyclist’s progress as they undertake a TT on a track of a known distance. Comprehensive data from the performance can be stored and the avatar can be replayed, serving as an opponent for the cyclist to race against in future trials. Performance from participants first experimental 4000 m TT was set as their baseline (BL). During the subsequent experimental trials participants were deceived into thinking they were to race an avatar representing their BL performance, but the actual avatar was racing at a power output that was either +2% or +5% higher than BL. The view seen by the cyclist was from behind the slower of the two avatars, meaning that they were able to monitor the performance of both, and could estimate the distance separating their current and previous performances. The only other information that could be seen on the projector screen was distance travelled. All other feedback was blinded from the participants. Participants were instructed to complete the distance as fast as possible, and were able to modulate power output through changes in cadence and use of an electronics gearing system. Throughout each trial expired air was analyzed breath-by-breath.

On completion of each trial, participants were asked to provide a global rating of perceived exertion using a 15-point scale [14] and to report their affective response to the whole trial using an 11-point feeling scale anchored from very good (+5) through neutral (0) to very bad (−5), which has previously been shown to provide valid ratings of affect following exercise [15]. Finger-tip capillary blood (20 μL) was sampled 2 min post-TT.

#### Aerobic and anaerobic contributions to total power output

Prior to each 4000 m TT, participants performed a standardized warm-up consisting of cycling for 5 min at 150 W and then a further 5 min at 70% of P_max_. Breath by breath $\dot{V}O_{2}$ and RER were measured continuously for the determination of gross mechanical efficiency (GME) during the 5 min of cycling at 70% of P_max_ using the equation *GME = P*_*TOT*_*/P*_*MET*_; where *P*_*MET*_ is the aerobic metabolic power, which can be calculated as:

$$P_{MET}=(\dot{V}O_{2}(4940\cdot RER+16040))/60$$ (1)

It was assumed that an RER greater than 1.0 was attributable to buffering, therefore in the calculation of metabolic work any RER values in excess of 1.0 were treated as if they equaled 1.0.

Aerobic (P_AER_) and anaerobic (P_AN_) contributions to total power output (P_TOT_) were calculated from P_TOT_, $\dot{V}O_{2}$, RER and GME. Oxygen uptake and RER measured during the TT were interpolated to 1 s values and time aligned with the P_TOT_ data. These data were then averaged into ten, 400 m ‘bins’, in order to facilitate between-trial analyses. To calculate P_AER_, aerobic metabolic power was multiplied by GME for each 400 m bin. Given that P_TOT_ is the sum of P_AER_ and P_AN_, P_AN_ was calculated as:

PAN=PTOT–PAER (2)

These methods, first described by Serresse et al. [16] and Serresse et al. [17], have subsequently been used by several researchers [18–23] and have recently been shown to be the most precise indirect estimate of anaerobic contribution to total power output during self-paced, simulated competition [24].

#### Data analysis

Statistical analyses were conducted using SPSS 16.0 (Chicago, Illinois, USA). Data are presented as mean ± SD. Differences in RPE, affect, peak $\dot{V}O_{2}$, peak HR, mean power output and time to completion between 4000 m trials were assessed using a one-way analysis of variance with repeated-measures. To investigate differences in the pacing strategy; P_TOT_, P_AN_ and P_AER_ were calculated for each 400 m section of each trial and evaluated using a factorial 3 x 10 (trial versus distance covered) repeated-measures ANOVA. Significant main effects were followed by Bonferroni post hoc tests. Statistical significance was accepted at *p*<0.05.

## Results

### Time-trial performance

**Fig 1:**
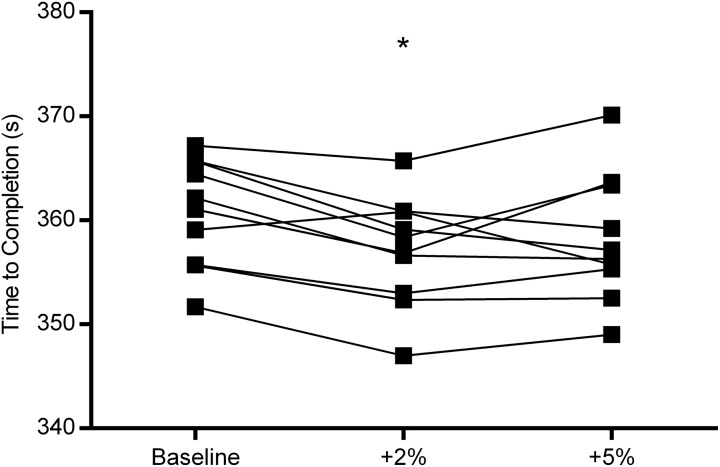
Individual 4000 m completion time during BL, +2% TT and +5% TT. *denotes a significantly faster completion time during +2% TT compared with BL (*p*<0.01).

Mean performance, cardiorespiratory and perceptual responses to 4000 m time trials are shown in Table 1. Time to completion was different between trials (F_2, 18_ = 6.665, *p* = 0.01) with the +2% TT finished more quickly (1.03%) than BL (*p*<0.01; 90% CI: -1.77 –-5.76 s; d = 0.7). There were no differences in completion time between either the +5% TT and +2% TT or the +5% TT and BL trials (*p*>0.05). Individual completion times for each trial are shown in Fig 1. Power output was also different between trials (F_2, 18_ = 5.560, p = 0.01). Pairwise comparisons revealed that power output was higher during the +2% TT compared with BL (*p* < 0.01; 90% CI: 3–14 W; d = 0.6) however there were no differences in power output between the +5% TT and +2% TT or +5% TT and BL (*p*>0.05).

**Table 1:** Performance, cardiorespiratory and perpetual responses to 4000 m time trials. Values shown are mean ± SD for the trial, except for blood lactate, RPE and affect for which post-trial values are shown.

	BL	+2% TT	+5% TT
Time to Completion (s)	360.8 ± 5.2	357.1 ± 5.3*	358.2 ± 6.1
Speed (km·h^-1^)	39.9 ± 0.6	40.3 ± 0.6*	40.2 ± 0.7
Power Output (W)	329.6 ± 14.5	338.1 ± 14.7*	335.3 ± 16.6
PAER (W)	274.9 ± 11.7	277.7 ± 10.4	278.6 ± 13.2
PAN (W)	54.7 ± 14.8	60.5 ± 14.9	56.8 ± 14.2
Cadence (RPM)	99.8 ± 17.5	98.8 ± 17.8	99.6 ± 18.6
$\dot{V}O_{2}$ (L.min^-1^)	4.38 ± 0.19	4.43 ± 0.17	4.44 ± 0.21
Peak $\dot{V}O_{2}$ (L.min^-1^)	4.73 ± 0.24	4.75 ± 0.28	4.77 ± 0.38
$\dot{V}CO_{2}$ (L.min^-1^)	5.04 ± 0.3	5.15 ± 0.33	5.13 ± 0.37
RER	1.14 ± 0.05	1.16 ± 0.06	1.15 ± 0.06
Heart Rate (BPM)	170 ± 11	173 ± 8	169 ± 9
Peak B[La] (mM)	12.48 ± 2.95	13.62 ± 3.07	12.95 ± 3.63
RPE	18.1 ± 0.9	18.3 ± 1.3	18.5 ± 1.2
Affect	-1.1 ± 2.5	-1.4 ± 2.4	-1.5 ± 2.4

BL, baseline 4000 m time trial; +2% TT, deception trial with false positive feedback equating to a 2% performance improvement; +5%, deception trial with false positive feedback equating to a 5% performance improvement

* = significantly different to BL (p < 0.01); PAER, aerobic contributions to total power output; PAN, anaerobic contributions to total power output; $\dot{V}O_{2}$, oxygen uptake; $\dot{V}CO_{2}$, carbon dioxide production; RER, respiratory exchange ratio; RPE, rate of perceived exertion.

**Fig 2:**
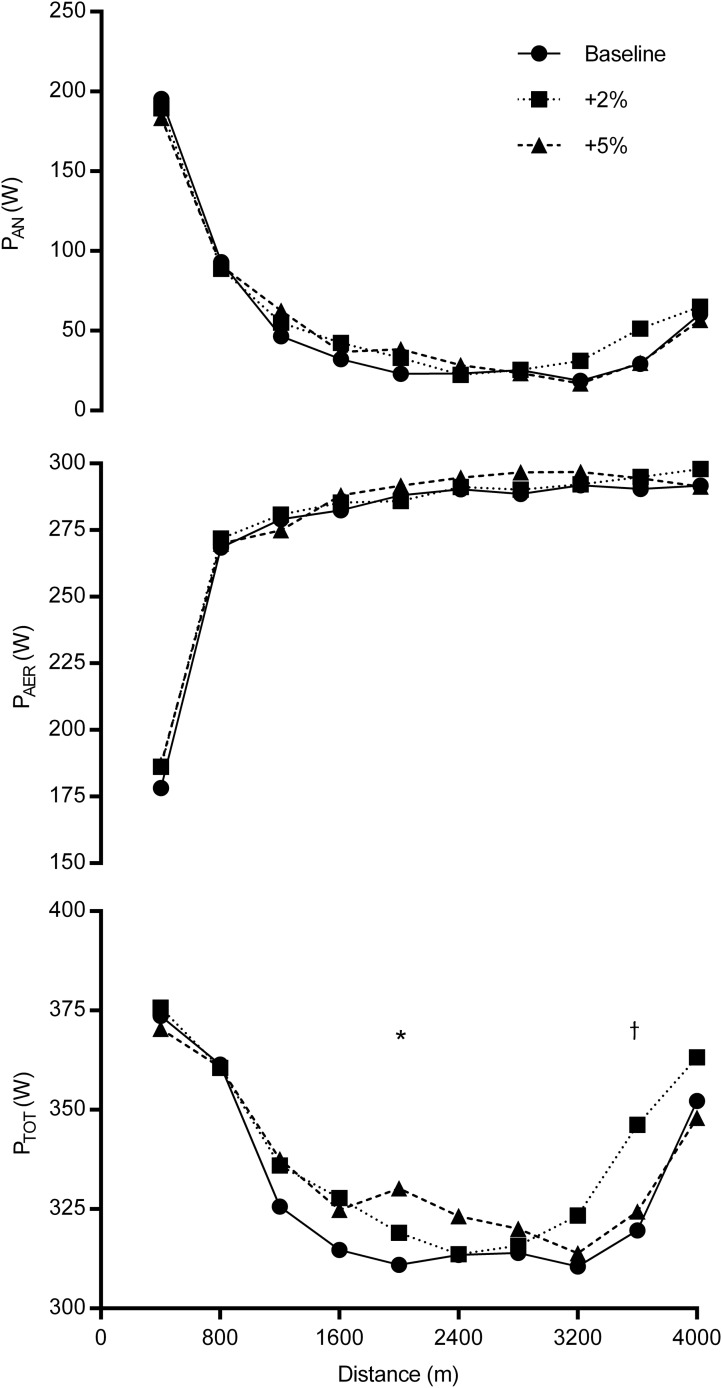
Serial patterns of P_TOT_, P_AN_ and P_AER_ during each 400 m section during BL, +2% TT and +5% TT. * denotes a significantly greater P_TOT_ during +5% TT compared with BL, and † denotes a significantly greater P_TOT_ during +2% TT compared with BL.

The serial patterns of P_TOT_, P_AN_ and P_AER_ are shown in Fig 2. No significant interactions were detected, however there were significant main effects for P_TOT_ at 2000 m (F_2, 18_ = 6.265, *p* = 0.01) and 3600 m (F_2, 18_ = 3.248, *p* = 0.04). Pairwise comparisons revealed that P_TOT_ was significantly greater in the +5% TT than BL at 2000 m (*p* = 0.04, 90% CI: 4–35 W; d = 0.8) and in the +2% TT than BL at 3600 m (*p* = 0.04; 98.3% CI: 0–54 W; d = 0.9). Pairwise comparisons revealed a trend towards higher P_AN_ during +2% TT compared with BL (*p* = 0.07; 90% CI: 2–42 W). There were no significant temporal variations in P_AER_ between trials, but the between-trial difference in P_AN_ at 3600 m approached significance (F_2, 18_ = 3.282, *p* = 0.06) with a trend towards higher P_AN_ during +2% TT compared with BL (90% CI: -6 to 50 W; d = 0.8).

### $\dot{V}E$, $\dot{V}O_{2}$, $\dot{V}CO_{2}$ and RER

Despite the small differences in power output, there were no discernible differences in the serial or mean trial respiratory responses between trials (*p* > 0.05).

### Affect, session RPE and peak blood lactate

There were no between-trial differences in RPE (*F*2, 18 = 0.643, *p* = 0.54), affect (*F*2, 18 = 0.701, *p* = 0.51) or Peak B[La] (*F*2, 18 = 2.02, *p* = 0.14)

## Discussion

This study aimed to examine the effects of deception on 4000 m cycling TT performance, when the magnitude of the deception represented either a small (+2%) or moderate (+5%) increase in power output. When the difference between anticipated and actual exercise intensity of the avatar was small, participants were able to reduce the amount of time taken to complete the TT. However, when the magnitude of the deception was equal to a moderate difference in 4000 m TT performance, participants did not demonstrate an improvement in performance compared to BL, although the performance was also not significantly different to +2% TT. These observations support the assertion that deception can elicit improvements in simulated TT performance and adds the novel finding that the ergogenic effect of deception is magnitude dependent. What magnitude of deception is most efficacious for performance and how it varies between different durations, intensities and modes of exercise remains unclear; however in this study, which manipulated 4,000 m cycling time trials, an increase in mean power output of 2% elicited an improved performance compared to baseline while an increase of 5% was equivocal.

The finding that participants were able to complete the +2% TT in a shorter amount of time is consistent with a previous study in our laboratory [6] and a more recent study [25] where a trend toward an improved performance compared to a baseline trial was also observed in a +2% TT (p = 0.08, CI -1.1 to 18.2 W). Despite the reduction in completion time and higher exercise intensity during +2% TT, there were no between-trial differences in either session RPE or affective response. This finding is consistent with Shei et al. [25] who also detected no significant difference in RPE between +2% TT and baseline trials, however it is inconsistent with our previous study where RPE was greater following the same degree of deception [6]. Whilst the reason for this inconsistency is unclear, Pohl et al. [26] have previously shown that psycho-physiological responses are largely determined by expectations; it is possible that in the present study the difference between actual and anticipated task difficulty during the +2% TT was sufficiently small to avoid detection, and that this masked changes in the symptoms of fatigue. Edwards and Polman [27] have proposed that small changes in pacing necessitating only minor modifications in homeostasis might operate at a subconscious level, in which case the RPE score which requires conscious integration of afferent and efferent feedback might not be affected. It is also possible that the existence of a competitor distracted the cyclists’ attentional focus, preventing an increase in RPE [28]. In either case, it would appear that the perception of effort was adjusted relatively with the pacing schema allowing more work to be completed for the same level of fixed effort [25].

Another reason for the discrepancy in RPE scores between studies might be due to the timing of the perceptual measurements. In the current study, a single post-exercise RPE score representing the whole trial was taken, whereas in the Shei et al. study [25] RPE was measured at the end of each 1,000 m of the 4,000 m TT. It is known that distance remaining affects RPE scores during exercise and that a single RPE score is also prone to variability as physiological exercise-related feedback diminishes following exercise cessation. It could be argued that measuring RPE only at trial cessation or at infrequent junctures might not adequately reflect subtle, momentary perceptions during exercise.

The present study used trained participants so it remains to be elucidated if elite-level riders would be able to detect a change in power output of this magnitude. Interoceptive sensitivity and physiological awareness are known to be developed through experience [29, 30] and therefore elite participants might be more able to readily detect the between-trial differences from physiological disturbances. Baron et al. [31] suggested that affective responses to exercise are influenced by a balance of the level of discomfort arising from physiological disturbances and the level of motivation, whereby when motivation is less than optimal, the chosen exercise intensity would be lower for the same affective load. Given that exercise intensity was greater during +2% it is likely that the level of motivation provided by the presence of competition was sufficient for participants to overcome the unpleasant sensations associated with the increased physiological disturbance. This notion is supported by the data of Shei et al. [25] who found no correlation between participants’ Self-Motivation Inventory Score and time to completion or any differences in self-motivation scores between +2% TT and BL trials.

The fact that a change in performance was found to be equivocal during the +5% TT might indicate this level of deception approximated to an upper limit regarding how much deception can be tolerated. It is likely that during +5% TT, the extent of peripheral disturbance associated with severe exercise such as depletion of phosphocreatine, and proton and myoplasmic phosphate accumulation, ultimately limited performance despite high levels of motivation during the task. Notably, 9 out of 10 participants (Fig 1) appear to improve in the +2% TT compared to BL, whereas 5 out of 10 participants in the +5% TT appear to demonstrate a worse performance compared to the +2% TT. However, it could also be argued that a significant difference between +5% TT and BL trials might have been observed with a larger sample size or that a deception beyond +2% but less than +5% might also improve performance. The current findings suggest an upper limit at or just beyond a 2% change in mean power output might exist, in trained cyclists, beyond which performance is no longer improved by deception.

Post-exercise RPE and affect responses were not significantly different in +5% TT compared to BL despite an increased PTOT being detected at 2000 m. However PTOT remained similar thereafter between the +5% TT and BL trials and so the post-trial perceptual measurements are perhaps reflective of this rather than the increased PTOT detected at 2,000 m. This would suggest that a decision was made to reduce exercise intensity during the +5% TT which served to ameliorate RPE and affect responses. This intuitive decision was likely based on participants’ levels of motivation, afferent feedback, progress of the avatars relative to one another and knowledge of the remaining trial distance. Renfree et al. [32] have postulated that a decision to change power output will depend upon the perceived benefits of doing so. In the present study, participants appear to have decided to proceed with a pacing strategy less likely to incur a greater degree of homeostasis disruption in the +5% TT, possibly in anticipation of avoiding physiological system failure (27).

The increased power output and speed during +2% TT was associated with a non-significant increase in B[La] (90% CI: 0.18–2.10 mmol· l^-1^) and P_AN_ compared to BL. It has been suggested that lactate is an afferent signaling agent involved in complex systems control of self-paced exercise [33] and ordinarily it would be expected that increases in B[La] would result in a decreased exercise intensity in order to maintain an appropriate surplus of anaerobic energetic supplies. However, these findings and our previous findings [6] suggest that under conditions of deception, participants are able to retrieve latent anaerobic resources that are not ordinarily accessible. The fact that a surplus of the body’s finite anaerobic energy supplies is set aside under normal conditions could be argued to be consistent with the underlying principles of the complex-systems model of fatigue, which is to preserve homeostasis [33–36].

A closer analysis of the race profiles shows how the provision of false feedback influenced the pacing strategies selected by these participants perhaps causing the different performance outcomes between the +5% TT and +2% TT trials. Specifically, participants increased their energy expenditure at ~2000 m during +5% TT and at ~3200 m during +2% TT. It was evident that in both conditions, participants had been able to match the performance of the pacer until these respective points. From video playback of each trial it was found that when the distance between the “deception” avatar and their “real-time” avatar began to widen, the participants increased their exercise intensity in an attempt to match the “deception avatar”, to what they believed to be their BL performance. This decision might be explained by the findings of a previous study in which participants responded to an opponent overtaking them by mobilising greater amounts of effort in an attempt to speed up and regain control of the race [37]. However, whilst participants were able to sustain the additional exercise intensity until completion of the +2% TT, exercise intensity reduced to BL levels during +5% TT between ~60–80% of the total distance. These findings are consistent with the results of a previous study [7] during which participants who had been led to believe that they were capable of greater levels of performance selected a higher-than-usual exercise intensity during the first 5000 m of a 20-km TT, but were unable to sustain the additional exercise intensity with power output markedly reduced later in the trial. The responses they observed were more marked than in the current study, however this can be explained by the markedly lower changes in power output and trial distance in our study (2–5% and 4,000 m) compared to their study (~15% and 20-km, [7]).

The distribution of PAN appears to have played a critical role in terms of an improved time to completion being observed relative to baseline in the +2% TT but not the +5% TT trial. Previously, Hettinga et al. [22] demonstrated that during 4000 m cycling trials, completed with either an evenly-paced, negatively-paced or positively-paced strategy, the distribution of the anaerobic energy contribution was altered rather than the distribution of the aerobic energy contribution. They also reported that improvements in participant performance coincided with a greater use of their anaerobic capacity. This assertion was supported by the findings of Stone et al. [6] who observed that when 4000 m TT performance improved during a trial incorporating a +2% TT condition compared to a BL condition, the participants demonstrated a greater PAN toward the end of the trial. The distribution of P_AER_ was not found to be different between the conditions. The findings of the present study also show that in 4000 m cycling TTs a change in power output around + 2% TT, affects the distribution of P_AN_ and the performance outcome but not P_AER_. It is plausible that the change in P_TOT_ in +5% TT compared to BL at 2000 m precluded a significant performance improvement being observed compared to BL, due to a proportion of the finite anaerobic capacity being used early on in the trial which could not then be accessed later in the trial.

## Conclusion

This is the first study to have shown that deception improves exercise performance during a 4000 m cycling TT when the magnitude of deception is equal to a small increase in exercise intensity while a moderate increase in exercise intensity was equivocal. The distinction observed between experimental conditions could be attributable to the distance remaining when participants first noticeably increased their exercise intensity in order to keep up with the avatar (~2000 m during +5% and ~800 m during +2%). With trained participants, it is possible that there is a metabolic reserve that can be utilized through an increased central motor drive, to produce a 2% increase in power output, when participants are deceived during a competitive, highly motivating task. However if the increase in power output is greater (5% or more) then overall performance might not be enhanced, potentially due to afferent feedback reflecting excessive changes in metabolic substrate utilization, such as the finite anaerobic capacity being utilized too soon (or rapidly) and a rapid build-up of metabolic waste products. This interoceptive feedback integrated with a sense of the distance still remaining and the competitor avatar moving away rapidly, influenced a conscious or subconscious decision to reduce power output part way through the exercise bout. These findings provide support for the notion that variations in pace are determined in an anticipatory manner by the product of how an athlete feels momentarily and how much of the event remains. As the endpoint approaches, the risk of an excessive disturbance to homeostasis progressively decreases. Therefore, participants can access latent anaerobic energy resources during the final stages to improve their performance, but not necessarily during the early or mid-portion of an event. The use of deception has implications for performance enhancement in athletes, and for our understanding of the mechanisms underpinning variations in pace during severe exercise.

## Supporting information

S1 Dataset (XLSX)
